# GATA4-targeted compound exhibits cardioprotective actions against doxorubicin-induced toxicity in vitro and in vivo: establishment of a chronic cardiotoxicity model using human iPSC-derived cardiomyocytes

**DOI:** 10.1007/s00204-020-02711-8

**Published:** 2020-03-17

**Authors:** S. Tuuli Karhu, Sini M. Kinnunen, Marja Tölli, Mika J. Välimäki, Zoltán Szabó, Virpi Talman, Heikki Ruskoaho

**Affiliations:** 1 Drug Research Program and Division of Pharmacology and Pharmacotherapy, Faculty of Pharmacy, University of Helsinki, P.O. Box 56, 00014 Helsinki, Finland; 2 Department of Pharmacology and Toxicology, Institute of Biomedicine, University of Oulu, Oulu, Finland; 3 National Heart and Lung Institute, Imperial College London, London, UK

**Keywords:** Doxorubicin, Cardiotoxicity, Human induced pluripotent stem cell-derived cardiomyocytes, Transcription factors, GATA4-targeted compounds, Cardioprotective

## Abstract

Doxorubicin is a widely used anticancer drug that causes dose-related cardiotoxicity. The exact mechanisms of doxorubicin toxicity are still unclear, partly because most in vitro studies have evaluated the effects of short-term high-dose doxorubicin treatments. Here, we developed an in vitro model of long-term low-dose administration of doxorubicin utilizing human induced pluripotent stem cell-derived cardiomyocytes (hiPSC-CMs). Moreover, given that current strategies for prevention and management of doxorubicin-induced cardiotoxicity fail to prevent cancer patients developing heart failure, we also investigated whether the GATA4-targeted compound 3i-1000 has cardioprotective potential against doxorubicin toxicity both in vitro and in vivo. The final doxorubicin concentration used in the chronic toxicity model in vitro was chosen based on cell viability data evaluation. Exposure to doxorubicin at the concentrations of 1–3 µM markedly reduced (60%) hiPSC-CM viability already within 48 h, while a 14-day treatment with 100 nM doxorubicin concentration induced only a modest 26% reduction in hiPCS-CM viability. Doxorubicin treatment also decreased DNA content in hiPSC-CMs. Interestingly, the compound 3i-1000 attenuated doxorubicin-induced increase in pro-B-type natriuretic peptide (proBNP) expression and caspase-3/7 activation in hiPSC-CMs. Moreover, treatment with 3i-1000 for 2 weeks (30 mg/kg/day, i.p.) inhibited doxorubicin cardiotoxicity by restoring left ventricular ejection fraction and fractional shortening in chronic in vivo rat model. In conclusion, the results demonstrate that long-term exposure of hiPSC-CMs can be utilized as an in vitro model of delayed doxorubicin-induced toxicity and provide in vitro and in vivo evidence that targeting GATA4 may be an effective strategy to counteract doxorubicin-induced cardiotoxicity.

**Electronic supplementary material:**

The online version of this article (10.1007/s00204-020-02711-8) contains supplementary material, which is available to authorized users.

## Introduction

Cardiotoxicity is a well-recognized devastating adverse outcome related to cancer therapy and can lead to long-term morbidity (Senkus and Jassem 2011). Its prevalence is increasing due to improved long-term survival of cancer patients. One of the most commonly used groups of anticancer drugs are the anthracyclines (e.g. doxorubicin, daunorubicin and idarubicin) which may cause acute cardiac damage that can be reversible, but more commonly cause late-onset toxicity that leads to heart failure. Anthracycline cardiotoxicity is dose-dependent with the heart failure incidence rates ranging from 0.14 to 48% (Conway et al. 2015). If baseline cardiotoxicity risk is high, a prophylactic cardioprotective treatment with angiotensin-converting enzyme inhibitors, angiotensin II receptor blockers, β-blockers and/or statins should be considered (Corremans et al. 2019; Zamorano et al. 2016). Other strategies to prevent left ventricular dysfunction and heart failure induced by anthracyclines include reduction in the cumulative dose and use of continuous infusions to decrease peak plasma levels, liposomal formulations and less toxic analogues of anthracyclines as well as FDA-approved cardioprotective agent dexrazoxane (Zamorano et al. 2016). Unfortunately, none of these strategies is efficacious enough to prevent a subset of cancer patients developing heart failure.

The exact mechanisms of anthracycline-induced cardiotoxicity are still unclear, but may involve oxidative stress, interaction with DNA topoisomerase II beta, calcium dysregulation, iron accumulation, mitochondrial damage, structural changes, and premature senescence as well as activation of immune system (Maejima et al. 2008; Octavia et al. 2012; Renu et al. 2018; Rochette et al. 2015; Zhang et al. 2012). Anthracyclines (Aries et al. 2004; Bien et al. 2007; Esaki et al. 2008; Kim et al. 2003; Kobayashi et al. 2006, 2010; Koka et al. 2010; Riad et al. 2008) along with ischemia (Suzuki et al. 2004) have been shown to induce apoptosis and downregulation of transcription factor GATA4 in the myocardium. Increased apoptosis has also been observed in adult cardiomyocytes in GATA4 knock-out mice and in mice with reduced GATA4 levels (Bisping et al. 2006; Oka et al. 2006) as well as in neonatal cardiomyocytes when GATA4 has been depleted by adenoviral antisense transcripts (Aries et al. 2004). In agreement with these findings, GATA4 overexpression in vivo by intramyocardial delivery of GATA4 adenoviral vector prevented myocardial infarction-induced apoptosis and adverse remodelling in rats (Rysä et al. 2010). Accordingly, overexpression of GATA4 in transgenic mice (Kobayashi et al. 2006) or by adenovirus-mediated gene transfer in vitro in neonatal cardiomyocytes and HL-1 cells prevented anthracycline-induced apoptosis (Aries et al. 2004; Kim et al. 2003; Kobayashi et al. 2006). The mechanisms of doxorubicin-induced decrease in GATA4 protein levels may involve downregulation of GATA4 gene expression (Park et al. 2011) or caspase-1-dependent depletion of GATA4 protein levels (Aries et al. 2014). On the other hand, GATA4 is a transcriptional regulator of the anti-apoptotic genes Bcl-xL (Aries et al. 2004; Kitta et al. 2003; Park et al. 2007) and Bcl-2 (Kobayashi et al. 2006) for which GATA4 binding activity and Ser-105 phosphorylation are required (Kobayashi et al. 2006). Overall, these findings demonstrate the significance of GATA4 for cell survival signalling.

One reason for the lack of full understanding of the mechanisms of doxorubicin cardiotoxicity may be that traditional preclinical models are not appropriate or sufficiently clinically relevant (Madonna et al. 2015). The translatability of results from in vitro and in vivo models to human is limited as these models are unable to reproduce the complex pathophysiology of human disease. For instance, animal models do not generally take into consideration ageing or comorbidities that aggravate drug-induced cardiotoxicities in clinical situations. Moreover, most in vitro studies have evaluated the effects of short-term high-dose doxorubicin treatments (Corremans et al. 2019). Additionally, due to interspecies differences, in vitro and in vivo animal models do not predict accurately the toxic effects on human heart. Thus, better experimental models of doxorubicin cardiotoxicity are needed to more appropriately simulate clinical circumstances as well as the actions of potential cardioprotective agents.

The present study had two main aims. First, we aimed to establish an in vitro model of long-term low-dose administration of doxorubicin utilizing human induced pluripotent stem cell-derived cardiomyocytes (hiPSC-CMs). Cell viability was studied with the 3-(4,5-dimethylthiazol-2-yl)-2,5-diphenyltetrazolium bromide (MTT) assay. High-content analysis (HCA) was used to study changes in DNA content, transcription factor GATA4 levels, expression of pro-B-type natriuretic peptide (proBNP), as well as caspase activation. Additionally, to compare different cardiomyocyte types, toxicity was studied in both hiPSC-CMs and primary neonatal rat ventricular myocytes (NRVMs). Second, as we have recently described a novel family of small-molecule compounds that affect the protein–protein interaction of transcription factors GATA4 and NKX2-5 and improve cardiac function in experimental models of myocardial infarction and hypertension (Ferreira et al. 2017; Kinnunen et al. 2018; Välimäki et al. 2017), we aimed to investigate if the lead compound 3i-1000 has cardioprotective potential against doxorubicin cardiotoxicity in vitro and in vivo.

## Materials and methods

### Reagents

Cell culture media, supplements and reagents were purchased from Gibco (Thermo Fisher Scientific, Paisley, UK). Bovine serum albumin (BSA), insulin–transferrin–sodium selenite media supplement, sodium pyruvate, pancreatin, 4′,6-diamidino-2-phenylindole (DAPI), and 3-(4,5-dimethylthiazol-2-yl)-2,5-diphenyltetrazolium bromide (MTT) were purchased from Sigma-Aldrich (Steinheim, Germany). Collagenase type 2 was purchased from Worthington Biochemical Corporation (Lakewood, New Jersey, USA). Growth Factor-Reduced Matrigel® was bought from Corning (Bedford, Massachusetts, USA) and gelatin from Merck Millipore (Darmstadt, Germany). Doxorubicin hydrochloride for in vitro studies as well as small-molecule inhibitors Y-27632, CHIR99021, and Wnt-C59 were acquired from Tocris Bioscience (Bristol, UK). Doxorubicin hydrochloride for the in vivo study was from Sigma-Aldrich. The small-molecule compound inhibiting GATA4 and NKX2-5 interaction (3i-1000; compound **3** in Välimäki et al. 2017), *N*-[4-(diethylamino)phenyl]-5-methyl-3-phenylisoxazole-4-carboxamide was purchased from Pharmatory LTD (Oulu, Finland). The purity of the compound (minimum > 95%) was determined by HPLC/MS and ^1^H NMR, and the possibility of aggregation of the compound was excluded (Välimäki et al. 2017). The primary antibodies used in immunofluorescence stainings were monoclonal rabbit anti-GATA4 (Cell Signaling Technology #36966), monoclonal mouse anti-proBNP (Abcam #ab13115), monoclonal mouse anti-α-actinin (Sigma-Aldrich #A7811), and polyclonal rabbit anti-cardiac troponin T (Abcam #ab45932). The secondary antibodies used were all purchased from Life Technologies (Eugene, Oregon, USA): Alexa Fluor 488 goat anti-mouse lgG (#A11029), Alexa Fluor 488 goat anti-rabbit lgG (#A11034), Alexa Fluor 647 goat anti-mouse lgG (#A21236), and Alexa Fluor 647 donkey anti-rabbit lgG (#A31573). eBioscience™ Brefeldin A Solution (1000X) and CellEvent™ Caspase-3/7 Green Detection Reagent were from Invitrogen (Carlsbad, California, USA). The primary antibodies used in western blotting were polyclonal rabbit anti-GATA4 (Santa Cruz Biotechnology #sc-9053), polyclonal rabbit anti-phospho-p38 (Cell Signaling Technology #9211), polyclonal rabbit anti-p38 (Cell Signaling Technology #9212), and monoclonal mouse anti-GAPDH (Merck Millipore #MAB374). The secondary HRP-linked antibodies anti-rabbit IgG (#7074) and anti-mouse IgG (#7076) were purchased from Cell Signaling Techonology. Quantitative real-time polymerase chain reaction (RT-PCR) oligos were from Sigma-Aldrich.

### Cell cultures

Long-term toxicity was studied in hiPSC-CMs. Acute toxicity was studied in both hiPSC-CMs and primary NRVMs. Cell cultures were maintained at 37 °C in a humidified atmosphere of 5% CO_2_. To investigate if the compound 3i-1000 is cardioprotective against doxorubicin-induced toxicity, the cells were exposed simultaneously to both doxorubicin and 3i-1000. The selection of doxorubicin concentrations was based on plasma concentrations detected in patients undergoing treatment (Creasey et al. 1976; Greene et al. 1983; Muller et al. 1993; Speth et al. 1987). The selection of 3i-1000 concentrations was based on previous studies investigating the efficacy and toxicity of the compound in vitro (Karhu et al. 2018; Välimäki et al. 2017). For compound exposures doxorubicin, 3i-1000, and equivalent vehicle dilutions were made separately in the growth medium. Compound exposures were started by aspirating the old growth media and adding first the media containing 3i-1000 (or equivalent volume concentration of dimethylsulfoxide; DMSO) to the cells. Cells were incubated at 37 °C for 10–15 min after which the medium containing doxorubicin (or equivalent volume concentration of DMSO) was also added to the cells. During long-term exposures, the media were replaced with fresh growth media (containing doxorubicin and/or 3i-1000) every 3–4 days.

### Human induced pluripotent stem cell-derived cardiomyocytes

The iPS(IMR90)-4 line (Yu et al. 2007) was purchased from WiCell (Madison, Wisconsin, USA). The stem cells were cultured in Essential 8™ medium (E8) on six-well plates coated with Matrigel^®^ (1:50). For passaging, the cells were dissociated with Versene^®^ and resuspended in E8 containing 10 µM ROCK inhibitor Y-27632. The cells were grown until 80–95% confluent. Cardiomyocytes were produced from hiPSCs using small-molecule induction, as described earlier (Burridge et al. 2014; Karhu et al. 2018). Differentiation was started by adding 6 µM CHIR99021 in RPMI 1640 medium supplemented with B-27 without insulin (RB-ins) to the cells (day 0). After 24 h, CHIR99021 was removed and replaced with fresh RB-ins (day 1). On day 3, the medium was changed to RB-ins containing 2.5 µM Wnt-C59 for 48 h. From day 5 to 11, the cells were maintained in RB-ins. To purify the cardiomyocyte cultures, on day 11 and 13, the cells were fed with RPMI 1640 without glucose with B-27 supplement. From day 15 onwards, the cells were maintained in RPMI 1640 supplemented with B-27 (RB + ins). Beating hiPSC-CMs were dissociated between days 15 and 17 by incubating them in cell dissociation solution containing 40% enzyme-free cell dissociation buffer, 40% RPMI 1640 and 20% trypsin–EDTA (final trypsin concentration 0.01%) for 7–8 min. Trypsin was inactivated with RB + ins supplemented with 10% foetal bovine serum (FBS). After centrifugation the cells were suspended in RB + ins with 10% FBS containing 10 µM ROCK inhibitor Y-27632 and seeded at 17,000–20,000 cells/well on gelatin-coated 96-well plates. In general, differentiation yielded almost pure (> 95%) cardiomyocyte cultures indicating high differentiation efficiency. In the experiments, only differentiation batches that were > 95% pure cardiomyocyte cultures were used. The cells were let to attach for 2 days, after which they were maintained in RB + ins (without FBS) for approximately 1 week before treatments. For compound exposures, RB + ins (without FBS) was used.

### Primary cardiomyocytes

Primary cultures of NRVMs were prepared from 1 to 3 day-old Wistar rats, as described earlier (Tölli et al. 2014). Animals were sacrificed by decapitation. Ventricles were dissected and cut into small pieces, which were then enzymatically digested by incubating them for 1–1.5 h at 37 °C under 600 rpm shaking conditions in a solution containing 100 mM NaCl, 10 mM KCl, 1.2 mM KH_2_PO_4_, 4.0 mM MgSO_4_, 50 mM taurine, 20 mM glucose, 10 mM 4-(2-hydroxyethyl)-1-piperazineethanesulfonic acid (HEPES), 2 mg/ml collagenase type 2, 2 mg/ml pancreatin, 100 U/ml penicillin, and 100 µg/ml streptomycin. The cell suspension was collected and centrifuged for 5 min at 160 × g. The supernatant and the top layer of the pellet were discarded and the isolated cardiac cells were resuspended in Dulbecco’s Modified Eagle Medium/Nutrient Mixture F-12 (DMEM/F12) supplemented with 10% FBS, 100 U/ml penicillin, and 100 µg/ml streptomycin. To reduce the number of contaminating non-myocytes (25–45% on day 3 after cell isolation), the cells were pre-plated onto cell culture flasks and let attach for 45–60 min in cell culture conditions. Unattached cells (enriched cardiomyocytes) were collected with the medium and seeded at 30,000–40,000 cells/well in gelatin-coated 96-well plates. Next day, the medium was changed to complete serum free medium (CSFM; DMEM/F-12 supplemented with 2.5 mg/ml BSA, 5 µg/ml insulin, 5 μg/ml transferrin, 5 ng/ml selenium, 2.8 mM sodium pyruvate, 0.1 nM triiodo-l-thyronine (T3), 100 U/ml penicillin, and 100 µg/ml streptomycin) for 24 h prior to compound treatments. For compound exposures, CSFM was used.

### Cell viability assay

The cells were exposed to doxorubicin and/or 3i-1000 for 2–21 days and cell viability was quantified with MTT assay (Mosmann 1983). MTT was added to the cells at a final concentration of 0.5 mg/ml followed by 2 h incubation in cell culture conditions. The medium was aspirated and formed formazan crystals were solubilized in DMSO. Absorbance was measured at 550 nm and absorbance at 650 nm was subtracted as background.

### Automated fluorescence microscopy and high-content analysis

The cells were exposed to doxorubicin and/or 3i-1000 for 1–14 days. For proBNP stainings, cells were additionally treated with Brefeldin A (1:1000) for 3 h prior to fixation. Alternatively, to study caspase activation, the cells were incubated with 7 µM caspase-3/7 detection reagent solution in phosphate-buffered saline (PBS) with 5% FBS for 60 min at 37 °C prior to fixation. The cells were fixed with 4% paraformaldehyde (PFA) for 15 min at room temperature (rt) and permeabilized with 0.1% Triton X-100 for 10 min. Non-specific binding sites were blocked with 4% FBS in PBS for 45 min at rt after which the cells were incubated with anti-GATA4 (1:400) or anti-proBNP (1:500) antibody. Additionally, a primary antibody against α-actinin (1:600) or cardiac troponin T (1:800) was used to identify myocytes. After a 60-min incubation with primary antibodies at rt, the cells were washed 3 × 5 min with PBS followed by a 45-min incubation with Alexa Fluor-conjugated secondary antibodies (1:200, with the exception of Alexa Fluor 647 anti-rabbit 1:250) and DAPI (1 µg/ml) at rt. The plates were imaged and analysed with CellInsight CX5 High-Content Screening Platform (Thermo Scientific) using a 10 × objective (Olympus UPlanFL N 10x/0.3). For quantification, the cells were first identified based on DAPI fluorescence, which defined the nuclear area. Non-myocytes were excluded based on absence of α-actinin/cardiac troponin T staining. The threshold for α-actinin/cardiac troponin T fluorescence intensity was set manually in each experiment to allow optimal exclusion of non-myocytes. The data were collected only from α-actinin/cardiac troponin T positive cells. The intensity of GATA4 staining was analysed within the nucleus. The intensity of proBNP staining was analysed in the perinuclear area defined by a 4-pixel ring around the nucleus. The threshold for proBNP positive cells was set manually in each experiment to adjust for minor variation in staining intensity. The intensity of fluorescent caspase-3/7 activity reporter was quantified within the nucleus. The threshold for caspase positive and caspase negative cells was also set manually in each experiment.

### Doxorubicin-induced cardiotoxicity in rats

Doxorubicin was administered i.p. to 7 weeks old male Sprague Dawley rats with average weight 216 g (range 189–245 g) at the dose of 1 mg/kg/day for 10 days (Hayward and Hydock 2007). Control animals received an equivalent volume of saline. Based on previous experiments, and due to its rapid metabolism (Kinnunen et al. 2018), the compound 3i-1000 was administered i.p. at the dose of 15 mg/kg two times a day for 2 weeks from week 7 to week 9. It was diluted to DMSO and administered to animals as 1:1 dilution in corn oil, control animals receiving DMSO with corn oil in equivalent volume. Transthoracic echocardiography was performed using the Vevo2100 high-frequency high-resolution linear array ultrasound system (FujiFilm VisualSonics, Toronto, Canada) and MS-250 transducer (13–24 MHz, axial resolution 75 μm, lateral resolution 165 μm) by a trained sonographer blinded to the treatments, as described previously (Jurado Acosta et al. 2017). Rats were sedated with isoflurane or anesthetized with ketamine (50 mg/kg, i.p.) and xylazine (10 mg/kg, i.p.). Using two-dimensional imaging, a short axis view of the left ventricle (LV) at the level of the papillary muscles was obtained and a two-dimensionally guided M-mode recording through the anterior and posterior walls of the LV were acquired. End-systolic and end-diastolic LV dimensions (ESD and EDD) as well as the thickness of the interventricular septum and posterior wall were measured from the M-mode tracings. LV fractional shortening (FS) and ejection fraction (EF) were calculated from the M-mode LV dimensions using Eqs. 1 and 2:

1 $${\text{FS}}\left( {\text{\% }} \right) = \{ ({\text{LVEDD}} - {\text{LVESD}})/{\text{LVEDD}}\} \times 100,$$

2 $${\text{EF}}\left( {\text{\% }} \right) = \{ ({\text{LVEDD}})^{3} - \left( {{\text{LVESD}}} \right)^{3} /{\text{LVEDD}}^{3} \} \times 100.$$

An average of three measurements of each variable were used. After echocardiographic measurements at 9 weeks, the terminally anesthetized animals were decapitated, hearts were excised, and the apex of left ventricle was immersed in liquid nitrogen and stored at − 70 °C for further analysis.

### RNA isolation from LV tissue and RT-PCR

The LV tissue was grinded in liquid nitrogen to powder, of which 1/3 was used for total RNA isolation using guanidine thiocyanate–CsCl method (modified from Cathala et al. 1983). Shortly, tissue powder was homogenised in 3 ml lysis buffer containing 4 M guanidium thiocyanate, 0.1 M Tris–HCl (pH 7.5), 7% β-mercaptoethanol and 1.0–2.0% Na-lauroylsarcosine with Ultra-Turrax^®^ (IKA^®^) and cell debris was pelleted for 10 min 3000 rpm (1791×*g*) 4 °C. The supernatant containing RNA was stored in − 80 °C for further treatment. RNA was isolated by ultracentrifugation overnight through a 5.7 M CsCl cushion at 4 °C. The resulting pellet was resuspended in lysis buffer and RNA was precipitated with 3 M sodium acetate (pH 5.2) (1/10 vol) and ice cold absolute ethanol (3 × vol) at least for 1 h at − 20 °C. The precipitated RNA was pelleted by centrifugation for 15–20 min 12,000 rpm (13,520×*g*) at 4 °C and washed with 70% ethanol in diethylpyrocarbonate (DEPC)-treated water followed by another centrifugation for 5–10 min as described above. The washing was repeated and RNA pellet was air dried before dissolving in DEPC-H_2_O. For quantitative RT-PCR analyses, cDNA was synthesised from total RNA with a First-Strand cDNA Synthesis Kit (GE Healthcare Life Sciences) following the manufacturer’s protocol. RNA was analysed by RT-PCR on an ABI 7300 sequence detection system (Applied Biosystems) using TaqMan chemistry. The results were quantified using ΔΔ*C*_*T*_ method and normalised to 18S RNA quantified from the same samples. The following sequences of the primers and the fluorogenic probe were used in assay: atrial natriuretic peptide (ANP, forward:GAAAAGCAAACTGAGGGCTCTG, reverse:CCTACCCCCGAAGCAGCT, probe: TCGCTGGCCCTCGGAGCCT) and B-type natriuretic peptide (BNP, forward: TGGGCAGAAGATAGACCGGA, reverse: ACAACCTCAGCCCGTCACAG, probe: CGGCGCAGTCAGTCGCTTGG).

### Protein extraction from LV tissue and western blot

Two thirds of the ground LV tissue was homogenised in 4 ml of lysis buffer (20 mM Tris, 10 mM NaCl, 0.1 mM EDTA, 0.1 mM EGTA, pH 8.0) containing protease and phosphatase inhibitors (1 mM β-glycerophosphate, 1 mM Na_3_VO_4_, 10 µg/ml leupeptin, 10 µg/ml pepstatin, 10 µg/ml aprotinin, 2 mM benzamidine, 1 mM phenylmethylsulfonyl fluoride, 50 mM sodium fluoride, 1 mM dithiothreitol). Of the homogenate, 0.8 ml was used for total protein extraction and the rest for nuclear protein extraction. Then, 0.2 ml of lysis buffer (100 mM Tris–HCl, 750 mM NaCl, 5 mM EDTA, 5 mM EGTA, 5% Triton X-100, 12.5 mM sodium pyrophosphate, 5 mM β-glycerophosphate, 5 mM Na_3_VO_4_, pH 7.5) was added into the total protein homogenate and vortexed for 30 s. After a 20-min centrifugation at 12,500 rpm (14,670×*g*) at 4 °C, the supernatant containing total proteins was collected. For nuclear protein extraction, the homogenate was divided into two sets, which were later combined. The homogenate was kept on ice for 15 min after which NP-40 was added at the final concentration of 0.6%. Sample was vortexed vigorously for 15 s and centrifuged for 30 s at 12,500 rpm (14,670×*g*) at 4 °C. The pellet was suspended into buffer (20 mM Hepes, 0.4 mM NaCl, 1 mM EDTA, 1 mM EGTA, pH 8.0) including inhibitors mentioned above and the parallel samples were combined. The samples were then vortexed vigorously for 45 min at 4 °C. After the final centrifugation at 12,500 rpm for 5 min at 4 °C, the supernatant containing nuclear proteins was collected. The protein concentrations were determined with the Bio-Rad Protein Assay. From each animal, 50 µg of total protein or 20 µg nuclear protein was resolved on 12% SDS-PAGE gel and transferred onto nitrocellulose membrane. After blocking the nonspecific background in 5% non-fat dry milk, the membranes were incubated with 1:1000 dilution of primary antibodies, except for anti-GAPDH, which was used at 1:100,000 dilution, at 4 °C overnight. After washing, the membranes were incubated for 1 h with an HRP-conjugated anti-rabbit or anti-mouse secondary antibody in 1:2000 dilution. The protein amounts were detected by enhanced chemiluminescence with ECL Plus reagents (RPN2132, Amersham Biosciences) followed by digitalization of chemiluminescence with Luminescent Imager Analyzer LAS-3000 (Fujifilm) and analysing with Quantity One software 4.6.6.Basic (Bio-Rad Laboratories). For a second immunoblotting, the membrane was stripped for 30 min at 60 °C in stripping buffer (0.16 M Tris–HCl, 6.5% SDS and 2.25% β-mercaptoethanol), blocked and probed with antibodies as described above.

### Statistics

In vitro results are expressed as mean from at least three independent experiments with error bars representing the standard error of the mean (SEM). For statistical analysis, non-normalized raw data was used. Statistical analyses were performed using IBM SPSS Statistics 24 software. Statistical significance was evaluated with randomized block ANOVA (experiment and treatment as factors) followed by Tukey’s HSD. In vivo results are expressed as mean with error bars representing SEM. For the first series of in vivo results, Welch’s *t* test was used to compare groups NaCl and DOX at each time point separately. For the second series of in vivo results, Levene’s test was used to analyse the equality of variances after which independent-samples *t* test was used to compare groups NaCl + V and DOX + V or DOX + V and DOX + 3i-1000. Differences at the level of *P* < 0.05 were considered statistically significant.

### Ethics

Animal experiments were carried out in accordance with the 3R principles of the EU directive 2010/63/EU governing the care and use of experimental animals, and following local laws and regulations [Finnish Act on the Protection of Animals Used for Scientific or Educational Purposes (497/2013, Government Decree on the Protection of Animals Used for Scientific or Educational Purposes (564/2013)]. The protocols were approved by the national Animal Experiment Board of Finland (ESAVI-2010-03964/Ym-23).

## Results

### Doxorubicin-induced chronic toxicity in hiPSC-CMs

**Fig. 1:**
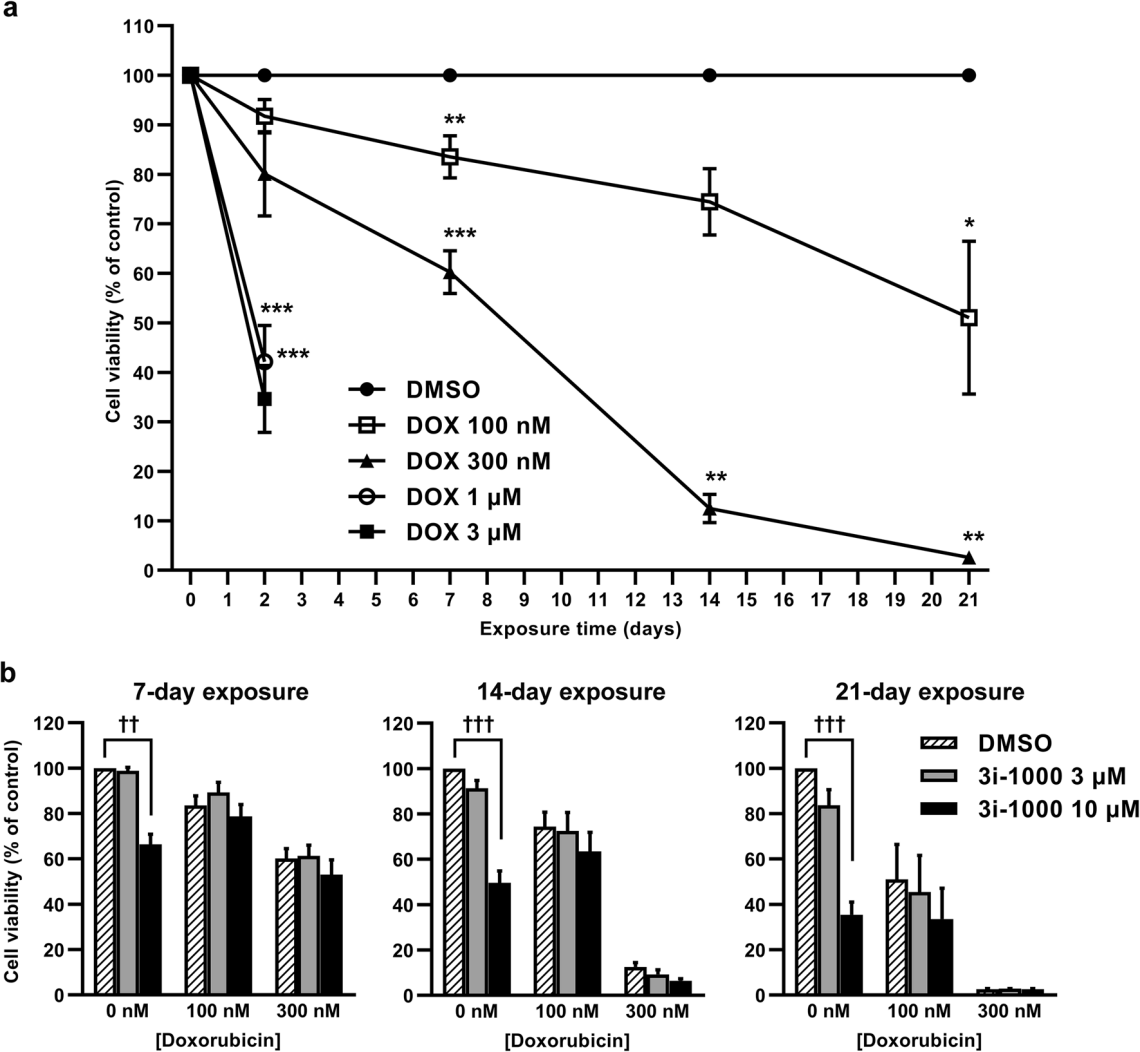
The effect of long-term doxorubicin (DOX) exposure on the viability of human induced pluripotent stem cell-derived cardiomyocytes (hiPSC-CMs). To study cell viability, the cells were exposed to DOX for 2–21 days after which the MTT assay was performed. **a** Cell viability after DOX treatment expressed as mean ± SEM (*n* = 4). **b** Cell viability after co-exposure to DOX and 3i-1000 expressed as mean + SEM (*n* = 4). ***P < 0.001 vs. DMSO; **P < 0.01 vs. DMSO; *P < 0.05 vs. DMSO; ^†††^*P * < 0.001 vs. control; ^††^*P * < 0.01 vs. control (randomized block ANOVA followed by Tukey’s HSD)

To study the long-term toxicity in vitro, hiPSC-CMs were exposed to doxorubicin for up to 21 days. Exposure to doxorubicin at concentrations of 1 µM and 3 µM markedly reduced hiPSC-CM viability already within 48 h (approximately 60%, *P * < 0.001; Fig. 1a). Treatment of hiPSC-CMs with 300 nM doxorubicin was less toxic but resulted in severe cytotoxicity within 21 days. On the other hand, a 14-day exposure to 100 nM doxorubicin induced only a modest 26% (*P* = 0.201) reduction in hiPSC-CM viability. Based on these results, doxorubicin at the concentration of 100 nM for 14 days was chosen for further HCA experiments to explore long-term toxicity. To investigate the effects of the small-molecule compound 3i-1000 in hiPSC-CMs, the cells were exposed to the compound alone or in combination with doxorubicin for 7, 14, and 21 days (Fig. 1b). In the MTT assay, 3i-1000 alone at 10 µM concentration reduced hiPSC-CM viability 34% (*P* = 0.001), 50% (*P * < 0.001) and 65% (*P * < 0.001) after 7, 14 and 21 days of exposure, respectively. At the concentration of 3 µM, the decrease was only 16% even after 21-day exposure. Moreover, 3i-1000 at 3–10 µM concentrations had no effect on doxorubicin-induced reductions in hiPSC-CM viability.

### Effects of doxorubicin and 3i-1000 on DNA content and GATA4 levels in hiPSC-CMs

**Fig. 2:**
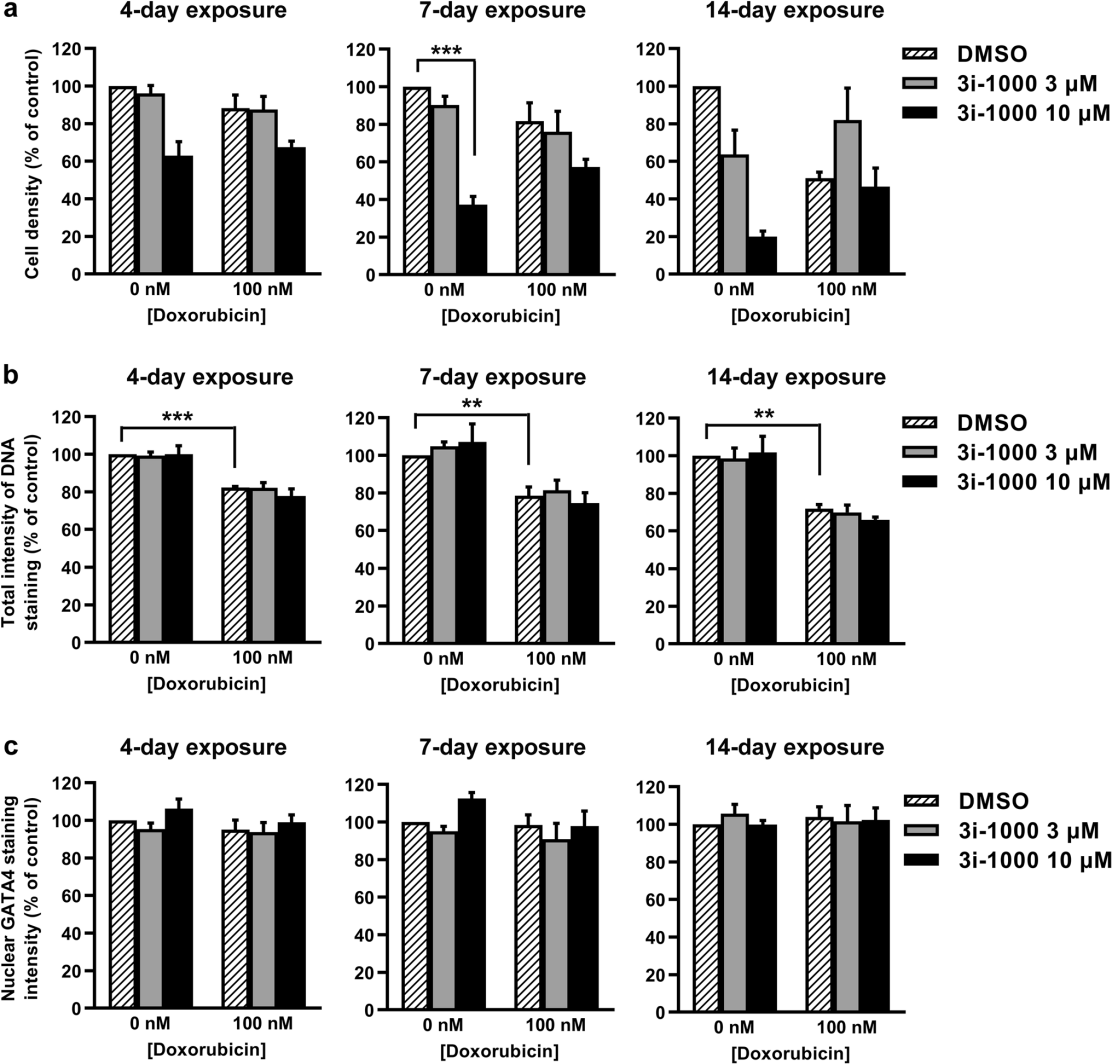
The effects of doxorubicin and 3i-1000 on DNA content and GATA4 levels in human induced pluripotent stem cell-derived cardiomyocytes (hiPSC-CMs) after long-term exposure. For high-content analysis, the cells were exposed simultaneously to 100 nM doxorubicin and 3i-1000 for 4, 7 or 14 days after which they were fixed and stained. Imaging and analysis was carried out using CellInsight High-Content Screening Platform. **a** Cell density. **b** Intensity of DNA staining. **c** Intensity of GATA4 staining in nucleus. The results are expressed as mean + SEM (*n* = 3–5). ***P  < 0.001 vs. control; **P  < 0.01 vs. control (randomized block ANOVA followed by Tukey’s HSD)

DAPI is a fluorescent dye that binds to A-T rich sequences of double-stranded DNA, thus the fluorescence depends on the amount of the DNA in the cells (Kapuscinski 1995). To evaluate the effect of doxorubicin and 3i-1000 on DNA content in hiPSC-CMs as well as hiPSC-CM density in culture, DAPI staining and HCA were utilized. Over long-term exposure, both doxorubicin and 3i-1000 decreased hiPSC-CM density in culture (Fig. 2a). After a 4-day exposure, doxorubicin-induced reduction in cell density was 12% compared to control, and after a 14-day exposure 49%. Additionally, a 14-day exposure to 10 µM 3i-1000 alone caused an 80% (*P* = 0.181) reduction in hiPSC-CM number compared to control, whereas at the concentration of 3 µM the decrease was 36%. A 4-day exposure to 100 nM doxorubicin decreased the average total intensity of DNA staining by 18% (*P * < 0.001) compared to control and after 14-day exposure this reduction was 28% (*P* = 0.003; Fig. 2b). We also measured variation in DNA staining intensity as an indication of DNA fragmentation leading to distribution of DNA fragments around nuclei (Darzynkiewicz et al. 2010; Doan-Xuan et al. 2013). Doxorubicin decreased the intranuclear variability of DNA staining intensity by 31% (*P * < 0.001), 37% (*P * < 0.001) and 44% (*P* = 0.009) after 4, 7 and 14-day exposures, respectively (Supplementary Fig. S1). The compound 3i-1000 had no significant effect on the total intensity of DNA staining or the intranuclear variability, either alone or in combination with doxorubicin. To elucidate the effect of doxorubicin and 3i-1000 on GATA4 levels in hiPSC-CMs, average GATA4 staining intensity in nucleus was analysed using HCA (Fig. 2c). Neither doxorubicin nor 3i-1000 had statistically significant effect on nuclear GATA4 staining even after a 14-day exposure.

### Effects of doxorubicin and 3i-1000 on proBNP expression in hiPSC-CMs

**Fig. 3:**
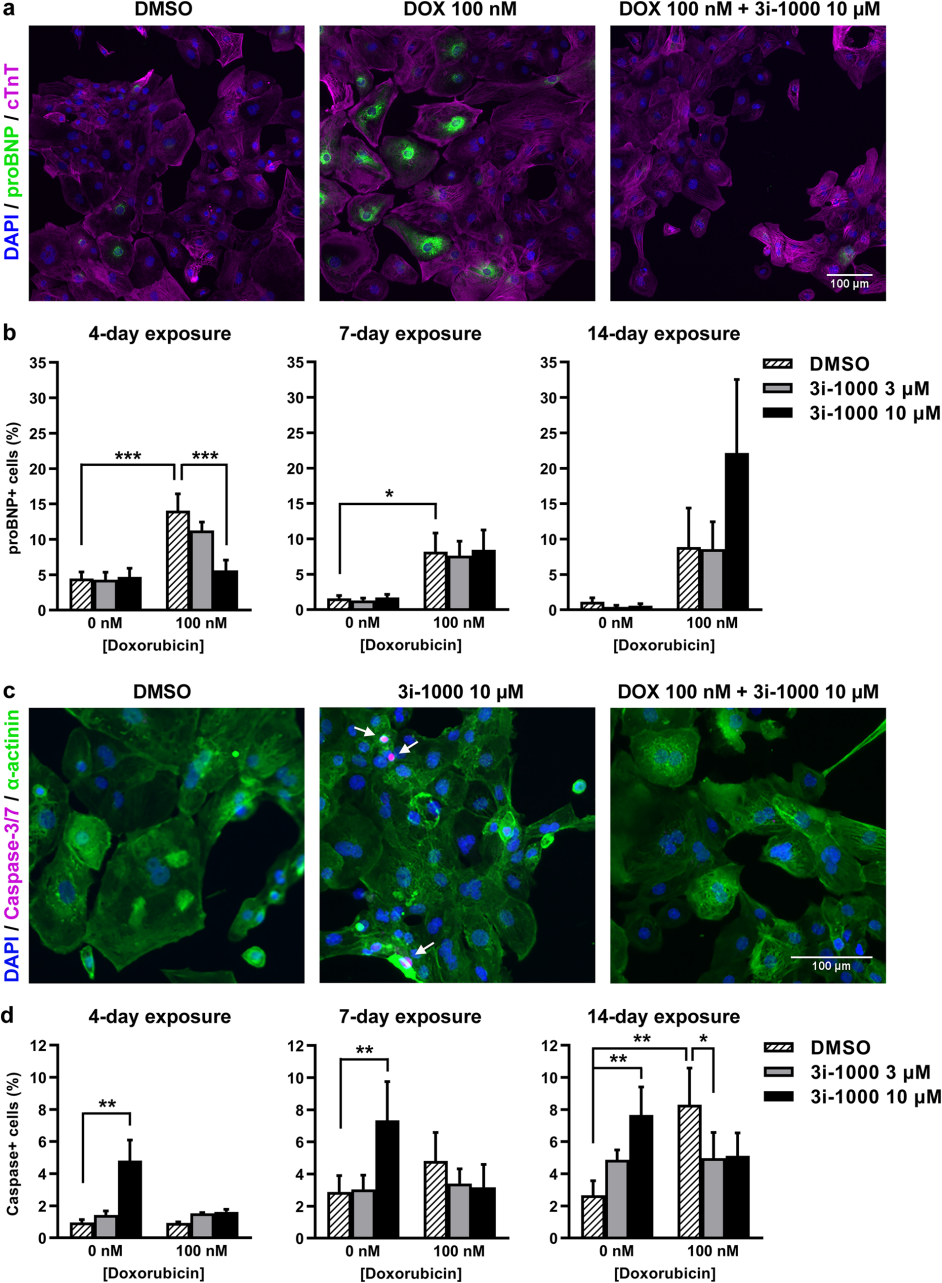
The effects of doxorubicin (DOX) and 3i-1000 on expression of pro-B-type natriuretic peptide (proBNP) and caspase activation in human induced pluripotent stem cell-derived cardiomyocytes (hiPSC-CMs) after long-term exposure. For high-content analysis, the cells were exposed simultaneously to 100 nM DOX and 3i-1000 for 4, 7 or 14 days after which they were fixed and stained. Imaging and analysis was carried out using CellInsight High-Content Screening Platform. **a** Representative images of proBNP staining after a 4-day exposure. **b** Proportion of proBNP positive hiPSC-CMs. **c** Representative images of caspase staining after a 4-day exposure. **d** Proportion of hiPSC-CMs positive for fluorescent caspase-3/7 activity reporter. Adjustments of individual colour channels to enhance brightness and contrast were made identically to all representative images. The results are expressed as mean + SEM (*n* = 3–4). ***P  < 0.001 vs. control; **P  < 0.01 vs. control; *P  < 0.05 vs. control (randomized block ANOVA followed by Tukey’s HSD). *cTnT* cardiac troponin T (colour figure online)

BNP is used for the diagnosis of heart failure and cardiac dysfunction (de Lemos et al. 2003; Ruskoaho 2003) and its synthesis in cardiomyocytes is induced by cellular stress such as mechanical stretch (Pikkarainen et al. 2003), hypoxia (Toth et al. 1994), and metabolic stress (Bistola et al. 2008), as well as various paracrine signals such as endothelin-1 (Bruneau et al. 1997) and cytokines (Ma et al. 2004). To evaluate the effect of doxorubicin and 3i-1000 on expression of the BNP precursor proBNP in hiPSC-CMs, proBNP staining and HCA were utilized. A 4-day exposure to 100 nM doxorubicin induced a 3.1-fold increase (*P* < 0.001) in the percentage of cardiomyocytes positive for proBNP compared to control (Fig. 3a, b), and this was paralleled by increased average intensity of proBNP staining in the perinuclear region (Supplementary Fig. S2). When the hiPSC-CMs were exposed simultaneously to 100 nM doxorubicin and 10 µM 3i-1000 for 4 days, the percentage of proBNP ^+^ cells decreased 60% (*P* < 0.001). Correspondingly, at the 3 µM concentration of 3i-1000, the decrease was 20%. On the other hand, percentage of proBNP + cells was similar in doxorubicin and doxorubicin plus 3i-1000 treated groups at day 7, as well as in doxorubicin and doxorubicin plus 3 µM 3i-1000 groups at day 14. However, when the cells were exposed simultaneously to doxorubicin (100 nM) and 10 µM 3i-1000 for 14 days, the percentage of proBNP  cells increased 19.3-fold compared to a 7.7-fold increase in cells exposed to 100 nM doxorubicin only (Fig. 3b).

### Effects of doxorubicin and 3i-1000 on caspase activation in hiPSC-CMs

To investigate the effect of doxorubicin and 3i-1000 on cell death, caspase activation was analysed using HCA. Doxorubicin at 100 nM concentration had no effect on the percentage of hiPSC-CMs positive for the fluorescent caspase-3/7 activity reporter after a 4-day exposure, but tended to increase after a 7-day exposure and produced a significant 3.1-fold increase (*P* = 0.001) in the percentage of cells with active caspase-3/7 after a 14-day exposure (Fig. 3d). The compound 3i-1000 alone at 10 µM concentration caused a 5.0-fold increase (*P* = 0.007) in cells positive for the caspase reporter after a 4-day exposure (Fig. 3c, d). Caspase-3/7 activity was significantly increased also after 7-day (2.5-fold, *P* = 0.007) and 14-day (2.9-fold, *P* = 0.003) exposures to 10 µM 3i-1000. At the 3 µM concentration of 3i-1000, the increase was 1.8-fold compared to control at day 14. Interestingly, when the cells were exposed to 10 µM 3i-1000 simultaneously with doxorubicin, the increases were only 1.7-fold, 1.1-fold and 1.9-fold (not statistically significant) compared to control at days 4, 7 and 14, respectively, indicating cardiomyocyte protective effect for 3i-1000 in hiPSC-CMs.

### hiPSC-CMs are more resistant to doxorubicin toxicity than primary cardiomyocytes

**Fig. 4:**
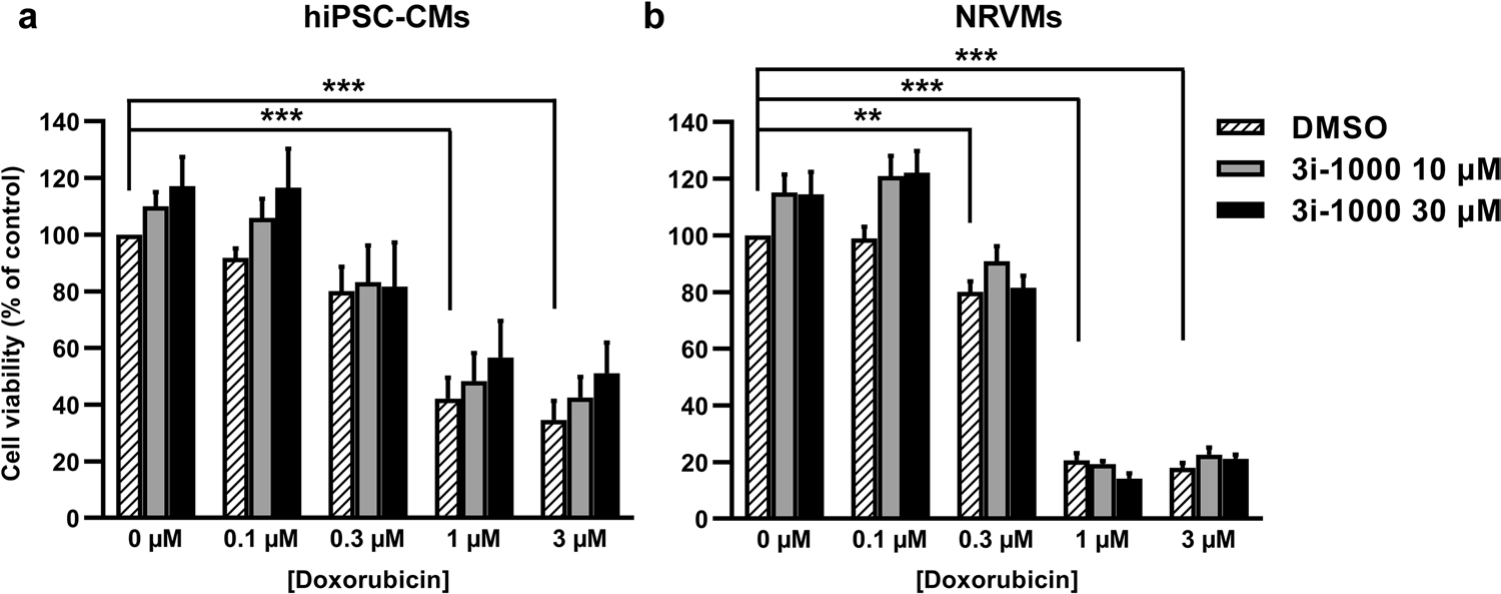
The effect of short-term doxorubicin exposure on the viability of human induced pluripotent stem cell-derived cardiomyocytes (hiPSC-CMs) and neonatal rat ventricular myocytes (NRVMs). To study cell viability, **a** hiPSC-CMs and **b** NRVMs were exposed simultaneously to doxorubicin and 3i-1000 for 48 h after which the MTT assay was performed. The results are expressed as mean + SEM (*n* = 3–4). ***P  < 0.001 vs. control; **P  < 0.01 vs. control (randomized block ANOVA followed by Tukey’s HSD)

To compare different cardiomyocyte types, hiPSC-CMs and NRVMs were exposed short-term to doxorubicin and 3i-1000 treatments. Short-term exposures were used because NRVMS, unlike hiPSC-CMs, cannot be cultured extended periods of time. In the MTT assay, a 48-h exposure to doxorubicin at 1 µM and 3 µM concentrations induced > 58% reductions (*P * < 0.001) in hiPSC-CM viability (Fig. 4a), whereas viability of NRVMs decreased by > 79% (*P * < 0.001; Fig. 4b) (Supplementary Fig. S3 shows IC_50_ values for doxorubicin in both cell types). A 48-h exposure to 100 nM doxorubicin had no substantial effect on the viability of either cardiomyocyte type, while 300 nM doxorubicin decreased the viabilities of both cell types by 20%. The compound 3i-1000 (at 10 µM and 30 µM concentrations) alone or in the presence of 100 nM doxorubicin tended to increase the viabilities of both cell types (approximately 20%).

**Fig. 5:**
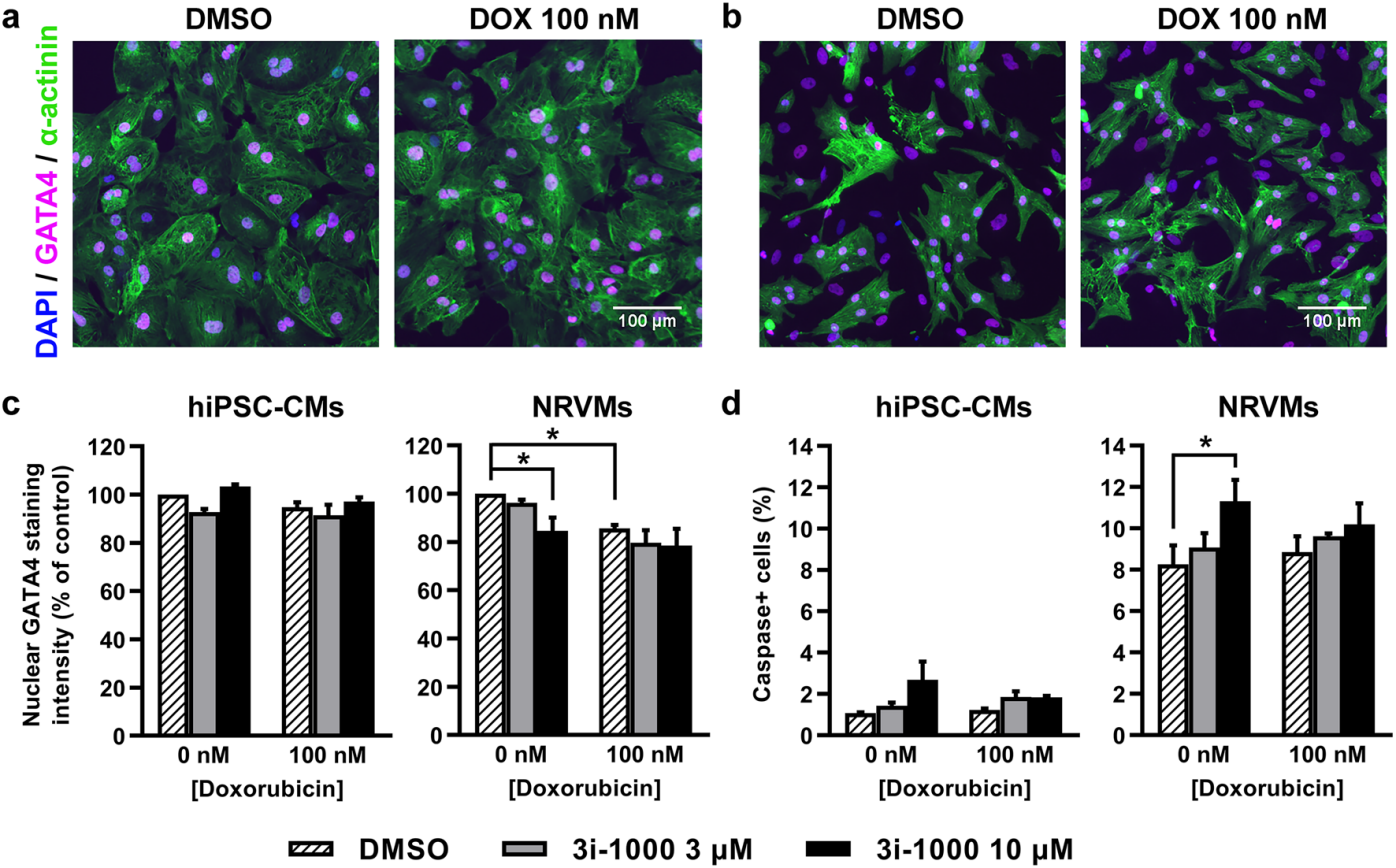
The effects of doxorubicin (DOX) and 3i-1000 on GATA4 levels and caspase activation in human induced pluripotent stem cell-derived cardiomyocytes (hiPSC-CMs) and neonatal rat ventricular myocytes (NRVMs) after short-term exposure. For high-content analysis, the cells were exposed simultaneously to 100 nM DOX and 3i-1000 for 24 h after which they were fixed and stained. Imaging and analysis was carried out using CellInsight High-Content Screening Platform. Representative images of GATA4 staining in **a** hiPSC-CMs and **b** primary NRVM cultures. **c** Average intensity of nuclear GATA4 staining. **d** Proportion of cardiomyocytes positive for fluorescent caspase-3/7 activity reporter. Adjustments of individual colour channels to enhance brightness and contrast were made identically to all representative images. The results are expressed as mean + SEM (*n* = 3–4). *P  < 0.05 vs. control (randomized block ANOVA followed by Tukey’s HSD) (colour figure online)

Based on HCA results (Fig. 5), a 24-h exposure to 100 nM doxorubicin induced a 14% decrease (*P* = 0.039) in the average intensity of GATA4 staining in NRVM nuclei, but not in hiPSC-CMs. Also, 3i-1000 decreased nuclear GATA4 staining intensity in NRVMs by 15% (*P* = 0.030). Moreover, a 24-h exposure to 100 nM doxorubicin had no effect on the percentage of cardiomyocytes positive for the fluorescent caspase-3/7 activity reporter in either cell type. However, a 24-h exposure to 10 µM 3i-1000 by itself induced a 2.5-fold non-significant increase in hiPSC-CMs and a 1.4-fold significant increase (*P* = 0.037) in NRVMs positive for the caspase reporter compared to DMSO, but not in the presence of 100 nM doxorubicin. It is also notable that in NRVM cultures the basal level of caspase + cardiomyocytes after 24-h exposure to 0.1% DMSO (on day 3 after cell isolation) was 8%, whereas in hiPSC-CM cultures the basal level of caspase ^+^ cells was no more than 1%.

### In vivo model of chronic doxorubicin toxicity

**Fig. 6:**
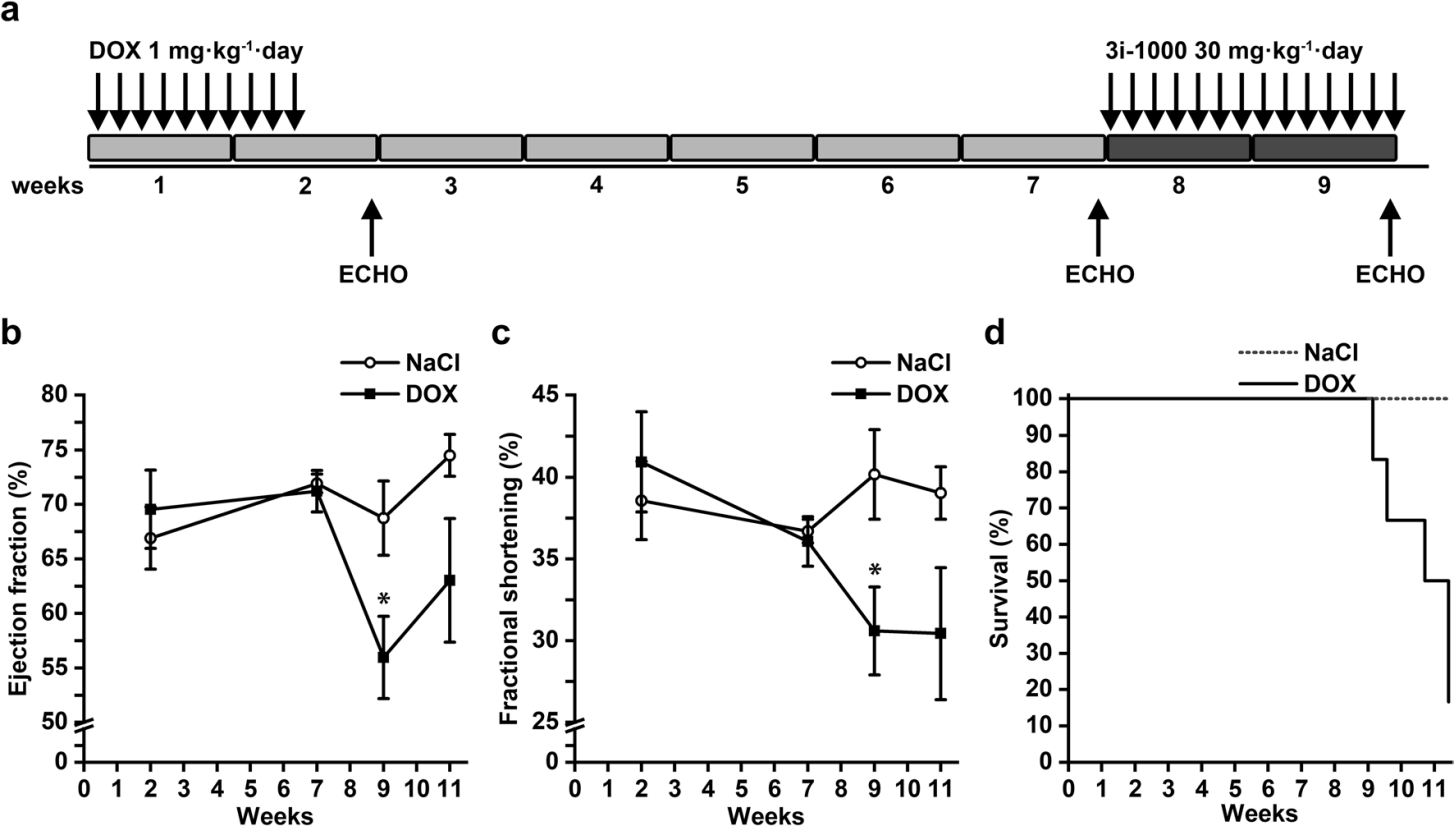
The in vivo animal model of doxorubicin (DOX) cardiotoxicity. The rats received saline or DOX 1 mg/kg/day for 10 days and were followed up to 11 weeks. **a** The experimental design of chronic DOX cardiotoxicity in rats. DOX was administered 1 mg/kg/day i.p. for 10 days. **b**, **c** Cardiac function was measured by echocardiography at 2, 7, 9 and 11 weeks. **d** The survival of the DOX-treated rats decreased quickly after 9 weeks. The results are expressed as mean ± SEM; *P  < 0.05 vs. control (Welch’s *t* test). Number of animals at 2, 7 and 9 week time points: NaCl = 3; DOX = 6, week 11 time point: NaCl = 3; DOX = 3. Panels **b–d** show data from animals that were not treated with 3i-1000. *ECHO* echocardiography

As the compound 3i-1000 showed cardioprotective effects in hiPSC-CMs in vitro, we next examined its effects on doxorubicin-induced cardiotoxicity in vivo. First, we carefully tested several rat and mice models (single 15 or 20 mg/kg dose of doxorubicin), as described in previous GATA4 in vivo cardioprotection studies (Kobayashi et al. 2006) and in various other studies in which doxorubicin has been shown to affect GATA4 levels (Aries et al. 2004; Bien et al. 2007; Esaki et al. 2008; Koka et al. 2010; Riad et al. 2008). However, under our experimental conditions, using high bolus dose of 15 mg/kg or 7.5 mg/kg/week three times i.p. had no effect on LV ejection fraction, instead doxorubicin induced acute diarrhoea and ascites, and serious weight loss (18–20%) was observed after 7 days in 2/3 of the rats. In our subsequent studies, we finally observed that the model developed for rats by Hayward and Hydock (2007), in which doxorubicin was administered at the dose of 1 mg/kg/day for 10 days (Fig. 6a), possessed many classical signs of doxorubicin-induced late-onset dilated cardiomyopathy, reflected as the decline in both LV ejection fraction and fractional shortening (Fig. 6b, c). Cardiac function was studied by echocardiography at 2, 7, 9, and 11 weeks (Fig. 6b, c). The cardiomyopathy as consequence of doxorubicin treatment started to develop after 7 weeks. At week 9, the LV ejection fraction in the saline group was 68.8 ± 3.4% (*n* = 3) and DOX group 55.9 ± 3.8% (*n* = 6) (*P* = 0.044), and also LV fractional shortening was lower in DOX-treated animals. After week 9, the survival of DOX-treated animals decreased quickly (Fig. 6d).

### Compound 3i-1000 restores cardiac function in doxorubicin-treated animals

**Fig. 7:**
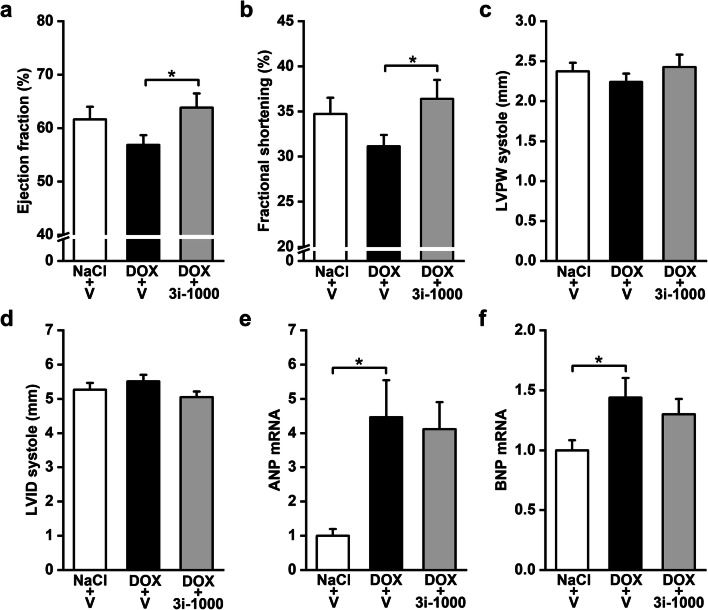
The effect of 3i-1000 on doxorubicin (DOX) cardiotoxicity in vivo. The rats received saline or DOX 1 mg/kg/day for 10 days and were then treated with compound 3i-1000 30 mg/kg/day or DMSO vehicle (V) for 2 weeks (weeks 8 and 9). **a–d** Left ventricular functional and structural changes measured by echocardiography at the end of the experiments. **e**, **f** mRNA extracted from left ventricles and measured by RT-PCR. The levels of transcripts were normalised to ribosomal 18S quantified from the same samples. The results are expressed as mean + SEM; *P < 0.05 (independent-samples *t* test). Number of animals: NaCl + V = 10; DOX + V = 9 (except for **e**, **f** DOX + V** = **8); DOX + 3i-1000 = 8. *LVPW* left ventricular posterior wall thickness, *LVID* left ventricular internal dimension

To study the effect of 3i-1000 on doxorubicin-induced cardiotoxicity in vivo, heart failure was first induced with doxorubicin treatment as described in Fig. 6a, and the compound 3i-1000 (or equal volume of vehicle) was injected i.p. at 30 mg/kg/day (the daily dose was divided in two portions) for 2 weeks during the weeks 8 and 9. Cardiac function was assessed by echocardiography at weeks 2, 7 and 9 (Supplementary Table S1). Interestingly, treatment with compound 3i-1000 significantly inhibited doxorubicin-induced cardiotoxicity by restoring the left ventricular EF (DOX plus 3i-1000 63.8 ± 2.6% vs. DOX 56.8 ± 1.8%, *P* = 0.041) and FS (DOX plus 3i-1000 36.4 ± 2.1% vs. DOX 31.2 ± 1.3%, *P* = 0.043) (Fig. 7a, b). There was no changes in left ventricular posterior wall thickness (LVPW) or internal dimension (LVID) (Fig. 7c, d). Doxorubicin-induced cardiac damage was associated with elevation of ANP and BNP mRNA expression and these increases in gene expressions were not significantly influenced by 3i-1000 treatment (Fig. 7e, f). We did not detect any significant changes on GATA4 protein levels by western blot analysis, but compound 3i-1000 inhibited the doxorubicin-induced decrease in phosphorylated-p38 protein levels (Supplementary Fig. S4).

## Discussion

Doxorubicin is a widely used chemotherapeutic agent but its clinical applications are limited by dose-dependent cardiotoxicity. Here, we have developed an in vitro model of long-term low-dose administration of doxorubicin utilizing hiPSC-CMs to more accurately mimic long-term doxorubicin dosing and late effects of cardiotoxicity in clinical practice. We exposed hiPSC-CMs to 100 nM doxorubicin for up to 21 days, which differs from the other hiPSC-CM-based models that have recently been used to study doxorubicin toxicity (Burridge et al. 2016; Chaudhari et al. 2016a, b; Louisse et al. 2017; Zhao and Zhang 2017). The concentration of doxorubicin was lower and the exposure time longer, which can be expected to more accurately model chronic dosing in human cancer patients and the cardiotoxicity that ensues. At the same time, it should be taken into account that extended exposure times with repeated medium changes may cause doxorubicin accumulation in the nuclei and associated cardiotoxicity (Kawai et al. 1997). The 100 nM concentration was chosen based on cell viability data evaluation. Micromolar concentrations of doxorubicin caused severe acute toxicity leading to considerable cell death already after 2 days. This acute toxicity was even more severe in NRVMs compared to hiPSC-CMs. Doxorubicin at 100 nM concentration, however, reduced cell viability over long-term exposure without causing excessive cell death. This model also allows the evaluation of efficacy of novel cardioprotective or restorative therapies on chronic cardiomyocyte toxicity in vitro.

Doxorubicin can intercalate with DNA, directly affecting transcription and replication, and leading to apoptosis of cancer cells (Yang et al. 2014). Doxorubicin-induced DNA damage and apoptosis contribute also to its cardiotoxicity (Arola et al. 2000; Lyu et al. 2007; Rochette et al. 2015; Zhang et al. 2012). Here we show that doxorubicin-induced decreases in cell number and viability and DNA content were associated with increased caspase-3/7 activity in the chronic in vitro cardiotoxicity model, confirming that caspase-dependent apoptosis contributed to cardiomyocyte death and thus cardiotoxicity. These findings further validate long-term low-dose exposure of hiPSC-CMs as a novel model of doxorubicin-induced cardiotoxicity.

To investigate doxorubicin cardiotoxicity in vivo*,* we subjected rats to a once a day regimen of doxorubicin for a period of 10 days and then followed up animals for 11 weeks. In previous studies of doxorubicin-induced cardiotoxicity, various animal models, doses and dosing regimens have been used (Aston et al. 2017). Here, our aim was to study the effect of the compound on cardiac function, and therefore, an animal model of doxorubicin-induced cardiotoxicity, in which the ejection fraction decreases, was necessary. The onset of cardiotoxicity was assessed by means of echocardiographic evaluation of cardiac function and natriuretic peptide measurements, both recommended also in clinical practice as diagnostic tools to detect myocardial toxicity (Zamorano et al. 2016). An important characteristic of the model used herein was the use of low doses of doxorubicin that resulted in delayed development of cardiac dysfunction, as shown by significant decrease in LV ejection fraction and fractional shortening only after week 7. This is in contrast to models that administered single or high doses of doxorubicin that rapidly damage the heart (Hayward and Hydock 2007). The dose of doxorubicin used here, however, was sufficient to activate left ventricular ANP and BNP gene expression, which is consistent with previous reports showing their expression to increase in response to cellular stress (Kinnunen et al. 1992, 1993; Ogawa et al. 1991; Toth et al. 1994). Regarding the clinical value, it is also noteworthy to point out that the studies with single high bolus dose of doxorubicin have been questioned, since they simulate acute cardiotoxicity (Corremans et al. 2019; Gianni et al. 2008). Therefore, a more relevant experimental design for the doxorubicin-induced cardiotoxicity would be the low-dose and repeated administrations, as this is used in clinics (Vejpongsa and Yeh 2014).

Regarding the translational value, it is important to compare also the doxorubicin dose used in the present experiments to the doses used in humans. In clinical use, doxorubicin is administered at the doses of 40–90 mg/m^2^ as at least 15 min-long intravenous infusions every third week (Vejpongsa and Yeh 2014). For paediatric patients and together with other chemotherapeutics lower doses are used. When a single dose of 12 mg/kg i.p. was administered to mice, doxorubicin plasma concentration was 60 ng/ml after 2 h and 20 ng/ml after 24 h (Johansen 1981). Correspondingly, in humans, 60 mg/m^2^ dose i.v. resulted doxorubicin plasma concentrations 480 ng/ml after 1 h and 40 ng/ml after 24 h (Barpe et al. 2010). Thus, in the present model in rats, the total cumulative dose of 10 mg/kg over 10 days roughly resembles a subchronic cardiotoxicity model. On the other hand, the cumulative dose 10 mg/kg in rats has been estimated to correspond to 400 mg/m^2^ in humans (80 kg, 183 cm) (Hayward and Hydock 2007). Moreover, in vitro concentrations 3 and 1 µM compare to the initial plasma levels of doxorubicin detected in patients after a bolus administration, whereas concentrations of 300 and 100 nM compare to the plasma levels that are reached within few hours after doxorubicin administration and are maintained by continuous infusion (Creasey et al. 1976; Greene et al. 1983; Muller et al. 1993; Speth et al. 1987).

Using these in vitro and in vivo models, we studied whether the GATA4-targeted compound 3i-1000 has cardioprotective effects on doxorubicin-induced cardiotoxicity. GATA4 is a member of the GATA family of zinc finger transcription factor, which was originally discovered as a regulator of cardiac development and subsequently identified as a major regulator of cardiac hypertrophy and cell survival (Pikkarainen et al. 2004; Suzuki 2011; Tremblay et al. 2018). Several hypertrophic stimuli directly regulate GATA4 DNA-binding and transcriptional activity in vitro (Hasegawa et al. 1997; Majalahti et al. 2007; Morimoto et al. 2000; Morisco et al. 2001; Kerkelä et al. 2002) and in vivo (Hautala et al. 2001; Majalahti et al. 2007). Moreover, mechanical stretch transiently increases GATA4 DNA-binding activity and transcript levels followed by increases in the expression of BNP, ANP, and skeletal α-actin genes (Pikkarainen et al. 2003). Interestingly, GATA4 overexpression alone induces hypertrophic myocardial cell growth and hypertrophic gene expression in GATA4 transgenic mice (Liang et al. 2001). Similarly, overexpression of GATA4 in cell culture by adenoviral gene transfer induces cardiomyocyte hypertrophy (Liang et al. 2001) and sarcomere reorganization as efficiently as endothelin-1 and phenylephrine (Charron et al. 2001). For stress-induced cardiac hypertrophic response, the GATA4 phosphorylation of Ser-105 has shown to be necessary (van Berlo et al. 2011).

Recently, we have reported the identification of small molecules that either inhibit or enhance the GATA4-NKX2-5 transcriptional synergy (Jumppanen et al. 2019; Välimäki et al. 2017). The most potent inhibitor of GATA4-NKX2-5 interaction, 3i-1000, had no influence on the baseline GATA4 proteins levels in NRVMs, whereas the phenylephrine-induced elevation in GATA4 Ser-105 phosphorylation was significantly inhibited by 3i-1000 (Kinnunen et al. 2018). Although the exact mechanisms of action remains to established, the compound 3i-1000 inhibits BNP transcription, and stretch-, endothelin-1- and phenylephrine-stimulated gene expression of ANP and BNP, as well as hypertrophic cell growth in cardiomyocytes while having no effect on GATA4 or NKX2-5 DNA binding or on the activity of protein kinases involved in the regulation of GATA4 phosphorylation (Välimäki et al. 2017; Kinnunen et al. 2018). Moreover, enhanced cardiac function in vivo in experimental models of myocardial infarction and hypertension has been observed (Kinnunen et al. 2018). Importantly, in our present experiments, the compound protected from doxorubicin-induced cardiac damage, as reflected by the restoration of LV ejection fraction and fractional shortening in doxorubicin-treated animals. Interestingly, improvement in cardiac function by 3i-1000 was not associated with the decrease in left ventricular ANP and BNP mRNA levels, suggesting a direct effect of doxorubicin on LV natriuretic peptide gene expression.

The compound 3i-1000 showed cardioprotective effects also in vitro. It attenuated doxorubicin-induced increase in proBNP expression in hiPSC-CMs after a 4-day exposure. Moreover, exposure of 3i-1000 at 3 µM and 10 µM concentrations attenuated doxorubicin-induced increase in caspase activation up to 14 days. The long-term exposures (up to 21 days), however, revealed toxic effects of 3i-1000 in cardiomyocytes. In our previous study (Karhu et al. 2018), the toxicity of eight compounds (3i-1000 and its derivatives) at concentrations ranging from 10 nM to 30 µM on the viability of eight different cell types were studied in detail. In these short-term experiments (24 h), 3i-1000 was non-toxic to cardiomyocytes (NRVMs, hiPSC-CMs), fibroblasts and H9c2 cardiac myoblasts. Interestingly, stem cells were very sensitive to detect toxicity of 3i-1000 and structure–toxicity analysis of all compounds revealed a characteristic dihedral angle in the GATA4-targeted compounds that may cause stem cell toxicity (Karhu et al. 2018).

Overall, our present in vitro results show that the protective effects of 3i-1000 on doxorubicin-induced cardiotoxicity are dependent on dose and treatment time, and also suggest distinct mechanisms of action for doxorubicin- and 3i-1000-induced cardiotoxicities. Doxorubicin had a direct effect on DNA content in cardiomyocytes, leading to caspase activation and apoptosis, whereas compound 3i-1000 had no direct effect on DNA content but increased caspase activity. Moreover, the present results not only show that 3i-1000 protected cardiomyocytes from doxorubicin-induced elevation of proBNP expression but also that doxorubicin protected cardiomyocytes from 3i-1000-induced caspase activation. Cell viability data at 7 and 14-day time points show the same effect: exposure to 3i-1000 at 10 µM concentration alone decreased hiPSC-CM viability, but this effect was attenuated with co-exposure to 100 nM doxorubicin. Thus, it is possible that targeting GATA4 with 3i-1000 may be detrimental to healthy cardiomyocytes in long-term. On the other hand, when cardiomyocytes are exposed to stressors (e.g. doxorubicin), co-treatment with 3i-1000 has protective effects in cardiomyocytes. Furthermore, together with our previous toxicological analysis of 3i-1000 and its derivatives, the present data supports further development of 3i-1000 derivatives.

Interestingly, in the present study doxorubicin had no effect on GATA4 protein levels either in vivo or in hiPSC-CMs even after long-term exposure. Statistically significant changes in GATA4 levels were detected only in NRVMs after short-term doxorubicin exposure. In previously published in vitro studies, in which doxorubicin was shown to decrease GATA4 mRNA and protein levels, doxorubicin concentrations were higher and exposure times shorter, ≤ 24 h (Aries et al. 2004, 2014; Kim et al. 2003; Kobayashi et al. 2006, 2010). Similarly, in the prior in vivo studies mice were treated with a single high-dose injection of doxorubicin (Aries et al. 2004; Kobayashi et al. 2006). Therefore, it is possible that the changes in GATA4 levels are related to short-term high-dose doxorubicin treatments. However, interspecies differences both in vitro and in vivo cannot be ruled out. Furthermore, the maturity level of the cells and the potential limitations that may entail should be considered when utilizing hiPSC-CMs. Although more investigations are needed in the future to fully understand the exact mechanisms of action of doxorubicin as well as the GATA4-targeted compound 3i-1000, our current results suggest that their mechanisms of action are not related to obvious changes in GATA4 protein levels.

Regarding preclinical drug development, our results highlight the importance of choosing an appropriate experimental model for compound testing already in early phases of drug discovery projects. The delayed toxicity of GATA4-targeted compound 3i-1000 demonstrates the significance of using longer exposure times in in vitro toxicity screening, which is possible when using hiPSC-CMs as these cells can be cultured for significantly longer periods of time compared to primary cardiomyocytes. Utilizing differentiated human cells also eliminates the influence of interspecies differences and helps to reduce the use of experimental animals. Furthermore, choosing a suitable model is a key element also in investigating the mechanism of doxorubicin cardiotoxicity as reflected by the lack of doxorubicin-induced GATA4 protein depletion in response to chronic low-dose treatments in hiPSC-CMs.

In summary, long-term exposure of hiPSC-CMs is a useful in vitro model to investigate the mechanisms of delayed doxorubicin-induced cardiotoxicity and novel cardioprotective therapies. The GATA4-targeted compound 3i-1000 exhibited cardioprotective potential in vitro as well as in vivo. Over chronic exposure the compound was, however, toxic to cardiomyocytes and hence further structural optimization is required to develop non-toxic derivatives.

## Electronic supplementary material

Below is the link to the electronic supplementary material.

Supplementary file1 (DOCX 421 kb)

## Data Availability

The datasets generated during and analysed during the current study are available from the corresponding author on reasonable request.
