# Supplementary material for: GATA4-targeted compound exhibits cardioprotective actions against doxorubicin-induced toxicity in vitro and in vivo: establishment of a chronic cardiotoxicity model using human iPSC-derived cardiomyocytes
Source: Arch Toxicol. 2020 Mar 17;94(6):2113–30. doi: 10.1007/s00204-020-02711-8 (PMC7303099; doi:10.1007/s00204-020-02711-8)
Supplement: Supplementary file 1 — Supplementary file1 (DOCX 421 kb) [file 204_2020_2711_MOESM1_ESM.docx]

***Supplementary information***

**GATA4-targeted compound exhibits cardioprotective actions against doxorubicin-induced toxicity *in vitro* and *in vivo*: establishment of a chronic cardiotoxicity model using human iPSC-derived cardiomyocytes**

S. Tuuli Karhu^+^, Sini M. Kinnunen^+^, Marja Tölli, Mika J. Välimäki, Zoltán Szabó, Virpi Talman, Heikki Ruskoaho*

**^*^**Corresponding author: Heikki Ruskoaho, Division of Pharmacology and Pharmacotherapy, Faculty of Pharmacy, University of Helsinki, P.O. Box 56, FI-00014 Helsinki, FINLAND, Tel. +358 50 4480772,

email:heikki.ruskoaho@helsinki.fi

^+^authors contributed equally to this work

**Contents:**

Supplementary Figures S1–S4, pages 2–4

Supplementary Tables S1, page 5

***Supplementary Figures***

***
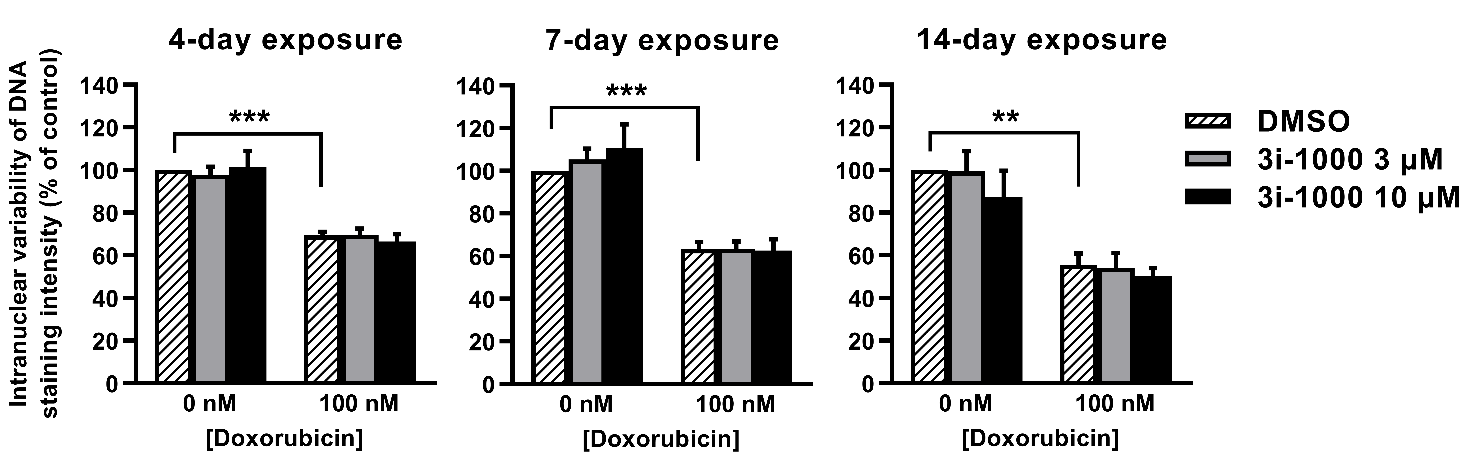
***

***Supplementary Fig. S1*** *The effects of doxorubicin and 3i-1000 on distribution of DNA in human induced pluripotent stem cell–derived cardiomyocytes (hiPSC-CMs) after long-term exposure. For high-content analysis, the cells were exposed simultaneously to 100 nM doxorubicin and 3i-1000 for 4, 7 or 14 days after which they were fixed and stained. Imaging and analysis was carried out using CellInsight High-Content Screening Platform. The quantifications for the average intranuclear variability in DNA staining intensity are expressed as mean + SEM (n=3–5). ***P˂0.001 vs. control; **P˂0.01 vs. control (randomized block ANOVA followed by Tukey’s HSD)*


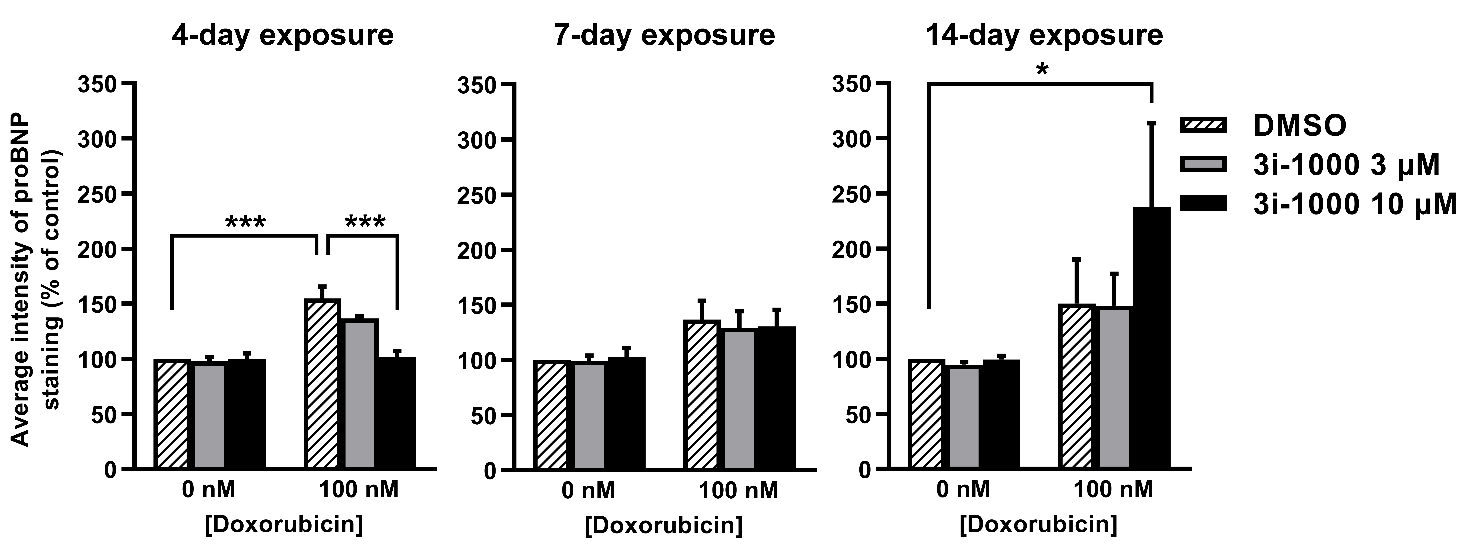


***Supplementary Fig. S2*** *The effects of doxorubicin and 3i-1000 on expression of pro-B-type natriuretic peptide (proBNP) in human induced pluripotent stem cell–derived cardiomyocytes (hiPSC-CMs) after long-term exposure. For high-content analysis, the cells were exposed simultaneously to 100 nM doxorubicin and 3i-1000 for 4, 7 or 14 days after which they were fixed and stained. Imaging and analysis was carried out using CellInsight High-Content Screening Platform. The quantifications for the average intensity of proBNP staining in the perinuclear area of hiPSC-CMs are expressed as mean + SEM (n=3–4). ***P˂0.001 vs. control; *P˂0.05 vs. control (randomized block ANOVA followed by Tukey’s HSD)*

*
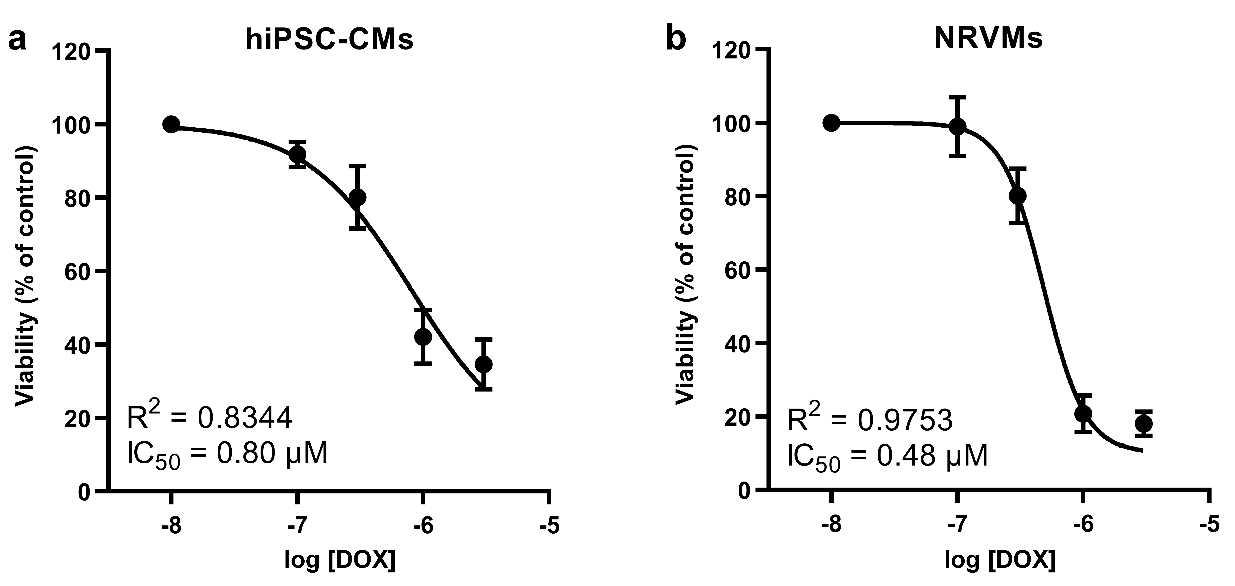
*

***Supplementary Fig. S3*** *The concentration response curves for doxorubicin (DOX) in* ***(a)*** *human induced pluripotent stem cell–derived cardiomyocytes (hiPSC-CMs) and* ***(b)*** *neonatal rat ventricular myocytes (NRVMs) after a 48-h exposure. Cell viability was determined with the MTT assay. Nonlinear regression curve fit and IC50 values were determined using GraphPad Prism 7. Results are expressed as mean ± SEM (n=4)*

***

***

***Supplementary Fig. S4*** *The effects of doxorubicin (DOX) and 3i-1000 on GATA4 and p38 mitogen activated protein kinase (MAPK) expression in the left ventricular tissue of rats. Nuclear and total proteins were extracted from left ventricles at the end of the experiments and analysed with western blot.* ***(a)*** *Representative western blot of GATA4 in nuclear fraction.* ***(b)*** *Quantified GATA4 protein levels adjusted to GAPDH.* ***(c)*** *Representative western blot of p38 MAPK and phosphorylated p38 MAPK in the total protein fraction.* ***(d–e)*** *Phophorylated p38 MAPK and total p38 MAPK quantifications from western blots adjusted to GAPDH.* ***(f)*** *Ratio of phosphorylated p38 MAPK to total p38 MAPK levels. The results are expressed as mean + SEM. Number of animals in p38 MAPK western blot quantifications* ***(b)****: NaCl+V=10; DOX+V=9; DOX+3i-1000=8, and in GATA4 western blot quantifications* ***(d–f)****: NaCl+V=6; DOX+V=6; DOX+3i-1000=4. *P<0.05 (independent samples t-test). V; vehicle (DMSO)*

***Supplementary Tables***

***Supplementary Table S1*** *The echocardiographic parameters of rats that received either NaCl or doxorubicin (DOX) 1 mg/kg/day for the first 10 days. Vehicle DMSO or the compound 3i-1000 (30 mg/kg/day, i.p.) was administered for two weeks from week 8 to 9. Echocardiographic measurements were done at 2 and 7 weeks and at the end of the experiment (9 weeks). Statistical comparison was made between DOX+DMSO vs. NaCl+DMSO and DOX+3i-1000 vs. DOX+DMSO groups at the 9-week time point (independent samples t-test). Number of animals in each time point: NaCl+V=10; DOX+V=9; DOX+3i-1000=8.* *A’; A peak, AET; aortic ejection time, E’; E peak, d; diastole, IVRT; isovolumetric relaxation time, LV; left ventricle, LVID; left ventricular internal diameter, LVPW; left ventricular posterior wall, s; systole*

|  | | **2 weeks** | | **7 weeks** | | **9 weeks** | |  |
| --- | --- | --- | --- | --- | --- | --- | --- | --- |
|  |  | **AVE** | **SEM** | **AVE** | **SEM** | **AVE** | **SEM** | **P** |
| **A' (mm/s)** | NaCl + DMSO | -48.99 | 6.52 | -65.44 | 3.41 | -27.75 | 2.93 |  |
|  | DOX+DMSO | -43.81 | 4.37 | -57.58 | 2.32 | -25.43 | 2.16 | 0.540 |
|  | DOX+3i-1000 | -45.02 | 5.19 | -54.80 | 5.30 | -28.10 | 1.58 | 0.345 |
| **E' (mm/s)** | NaCl + DMSO | -45.82 | 10.12 | -80.78 | 4.96 | -36.39 | 2.87 |  |
|  | DOX+DMSO | -42.07 | 5.37 | -72.27 | 2.96 | -30.31 | 3.33 | 0.182 |
|  | DOX+3i-1000 | -33.33 | 5.23 | -69.41 | 5.94 | -32.95 | 2.18 | 0.528 |
| **A'/E'** | NaCl + DMSO | 0.93 | 0.16 | 1.24 | 0.05 | 1.40 | 0.12 |  |
|  | DOX+DMSO | 0.99 | 0.11 | 1.27 | 0.06 | 1.23 | 0.12 | 0.337 |
|  | DOX+3i-1000 | 0.72 | 0.04 | 1.33 | 0.14 | 1.20 | 0.12 | 0.864 |
| **AET (ms)** | NaCl + DMSO | 55.16 | 2.23 | 59.01 | 1.66 | 78.23 | 2.70 |  |
|  | DOX+DMSO | 50.90 | 3.66 | 60.86 | 1.75 | 81.67 | 3.03 | 0.408 |
|  | DOX+3i-1000 | 55.69 | 2.42 | 59.56 | 0.90 | 83.73 | 5.15 | 0.728 |
| **IVRT (ms)** | NaCl + DMSO | 18.45 | 1.05 | 17.67 | 0.87 | 27.78 | 1.11 |  |
|  | DOX+DMSO | 21.64 | 0.52 | 18.27 | 0.97 | 32.87 | 2.45 | 0.060 |
|  | DOX+3i-1000 | 21.24 | 0.57 | 21.19 | 0.62 | 30.26 | 1.30 | 0.362 |
| **Cardiac Output (ml/min)** | NaCl + DMSO | 86.33 | 2.64 | 87.60 | 3.15 | 54.21 | 2.28 |  |
|  | DOX+DMSO | 75.05 | 2.50 | 82.04 | 2.34 | 40.54 | 2.44 | 0.001 |
|  | DOX+3i-1000 | 73.51 | 4.15 | 76.19 | 3.56 | 50.97 | 2.40 | 0.008 |
| **Ejection Fraction (%)** | NaCl + DMSO | 68.99 | 1.89 | 68.33 | 1.69 | 61.62 | 2.40 |  |
|  | DOX+DMSO | 66.92 | 2.06 | 62.77 | 1.33 | 56.85 | 1.81 | 0.138 |
|  | DOX+3i-1000 | 66.82 | 1.49 | 65.70 | 1.39 | 63.84 | 2.63 | 0.041 |
| **Fractional Shortening (%)** | NaCl + DMSO | 40.41 | 1.60 | 39.88 | 1.36 | 34.73 | 1.79 |  |
|  | DOX+DMSO | 38.56 | 1.58 | 35.43 | 0.97 | 31.15 | 1.26 | 0.128 |
|  | DOX+3i-1000 | 38.39 | 1.18 | 37.60 | 1.04 | 36.41 | 2.10 | 0.043 |
| **Heart Rate (bpm)** | NaCl + DMSO | 367.26 | 8.03 | 360.45 | 11.85 | 249.89 | 4.90 |  |
|  | DOX+DMSO | 365.28 | 10.09 | 355.47 | 4.35 | 199.67 | 10.54 | 0.000 |
|  | DOX+3i-1000 | 348.48 | 10.37 | 345.40 | 6.60 | 229.49 | 7.92 | 0.043 |
| **LV Mass (mg)** | NaCl + DMSO | 809.59 | 32.83 | 994.48 | 25.70 | 931.53 | 33.13 |  |
|  | DOX+DMSO | 764.67 | 19.76 | 974.33 | 26.84 | 878.57 | 30.43 | 0.259 |
|  | DOX+3i-1000 | 877.11 | 40.05 | 1044.27 | 36.40 | 923.04 | 58.29 | 0.495 |
| **Stroke Volume (µl)** | NaCl + DMSO | 235.52 | 7.04 | 243.65 | 7.28 | 216.89 | 7.67 |  |
|  | DOX+DMSO | 206.22 | 7.43 | 231.10 | 7.36 | 202.95 | 5.74 | 0.171 |
|  | DOX+3i-1000 | 210.78 | 9.39 | 220.51 | 8.86 | 222.79 | 9.74 | 0.091 |
| **Volume; d (µl)** | NaCl + DMSO | 343.06 | 12.19 | 358.18 | 12.72 | 353.99 | 11.33 |  |
|  | DOX+DMSO | 310.05 | 12.73 | 368.29 | 8.73 | 359.70 | 14.46 | 0.758 |
|  | DOX+3i-1000 | 315.46 | 12.37 | 336.78 | 15.19 | 349.49 | 9.35 | 0.573 |
| **Volume; s (µl)** | NaCl + DMSO | 107.54 | 8.95 | 114.53 | 9.05 | 137.10 | 11.82 |  |
|  | DOX+DMSO | 103.83 | 9.31 | 137.19 | 5.73 | 156.74 | 11.86 | 0.258 |
|  | DOX+3i-1000 | 104.68 | 5.97 | 116.27 | 8.84 | 126.69 | 10.32 | 0.078 |
| **LVID; d (mm)** | NaCl + DMSO | 7.95 | 0.11 | 8.06 | 0.12 | 7.95 | 0.12 |  |
|  | DOX+DMSO | 7.65 | 0.16 | 8.21 | 0.10 | 8.04 | 0.17 | 0.661 |
|  | DOX+3i-1000 | 7.62 | 0.11 | 7.88 | 0.16 | 7.90 | 0.09 | 0.483 |
| **LVID; s (mm)** | NaCl + DMSO | 4.82 | 0.16 | 5.10 | 0.14 | 5.27 | 0.20 |  |
|  | DOX+DMSO | 4.75 | 0.19 | 5.40 | 0.07 | 5.51 | 0.19 | 0.380 |
|  | DOX+3i-1000 | 4.74 | 0.12 | 4.99 | 0.17 | 5.05 | 0.17 | 0.088 |
| **LVPW; d (mm)** | NaCl + DMSO | 1.55 | 0.06 | 1.61 | 0.04 | 1.60 | 0.07 |  |
|  | DOX+DMSO | 1.55 | 0.06 | 1.56 | 0.05 | 1.46 | 0.08 | 0.210 |
|  | DOX+3i-1000 | 1.73 | 0.07 | 1.78 | 0.08 | 1.57 | 0.10 | 0.397 |
| **LVPW; s (mm)** | NaCl + DMSO | 2.53 | 0.09 | 2.51 | 0.07 | 2.37 | 0.11 |  |
|  | DOX+DMSO | 2.41 | 0.10 | 2.33 | 0.10 | 2.24 | 0.10 | 0.388 |
|  | DOX+3i-1000 | 2.65 | 0.11 | 2.61 | 0.12 | 2.43 | 0.16 | 0.330 |
